# The moderating effect of perceived hope in the relationship between anxiety and posttraumatic growth during the Russian-Ukrainian war

**DOI:** 10.3389/fpsyg.2024.1440021

**Published:** 2024-08-02

**Authors:** Alena Slezackova, Tatiana Malatincova, Katarina Millova, Miroslav Svetlak, Andreas M. Krafft

**Affiliations:** ^1^Department of Medical Psychology and Psychosomatics, Faculty of Medicine, Masaryk University, Brno, Czechia; ^2^Department of Psychology, Faculty of Arts, University of Ostrava, Ostrava, Czechia; ^3^Institute of Systemic Management and Public Governance, University of St. Gallen, St. Gallen, Switzerland

**Keywords:** perceived hope, posttraumatic growth, anxiety, Russian-Ukrainian war, mental health, psychological well-being

## Abstract

**Introduction:**

This study examines the relationships between perceived hope, posttraumatic growth, well-being, anxiety, and perceived threat of the Russian-Ukrainian War (RUW) in the Czech adult population. Drawing on the evidence of posttraumatic growth (PTG) amidst crisis, we hypothesized that perceived hope moderates the effects of perceived threat of war and anxiety on PTG.

**Methods:**

Data were obtained from 1,000 Czech respondents via an online questionnaire ten months post-invasion. The form included measures of posttraumatic growth, perceived hope, well-being, anxiety and depression, and perceived threat of war.

**Results:**

Our findings reveal that perceived hope acted as a moderator enhancing the positive effects of perceived threat and anxiety on PTG. However, perceived hope did not significantly moderate the direct effects of perceived threats and anxiety on well-being.

**Discussion:**

This study highlights the significant role of hope amidst adversity and underscores its potential as a target for interventions aiming to foster PTG in populations who navigate traumatic experiences. Furthermore, it advocates for continued exploration of the factors interacting to enhance well-being and facilitate PTG in affected communities.

## 1 Introduction

Over the past few years, the world’s societies have faced multiple challenges, which often arrived simultaneously or in close succession. The impacts of the COVID-19 pandemic have been combined with those of climate change, shortages of natural resources, and armed conflicts, affecting the psychological well-being and mental health of the general population (Barchielli et al., 2022). Comprehensive studies have demonstrated a profoundly negative impact of the COVID-19 pandemic on physical, emotional, and social dimensions of well-being across all affected countries (Fatahi et al., 2021; O'Connor et al., 2021; Zhang et al., 2021; de Girolamo et al., 2022). A study on large multi-country cohorts showed that almost one-third to one-half of the population had reported mental health issues during this global health crisis (Plomecka et al., 2021). Even before the traumas associated with the pandemic subsided, many people, especially across Europe, abruptly found themselves facing yet another tragic event: The Russian armed invasion of Ukraine, which began as another distressing event. The Russian armed invasion of Ukraine, which started in February 2022 and developed into a long-term armed conflict, has resulted in widespread destruction, refugee flows, loss of lives, and profound psychological distress for those directly affected. The Russian-Ukrainian war (RUW) has had significant political, socioeconomic, and health impacts in multiple countries apart from Russia and Ukraine and contributed to economic instability, increased tensions, and feelings of insecurity and threat across the European region (Sheather, 2022; The Lancet Regional Health–Europe, 2022; Vuorio et al., 2022; Zaliska et al., 2022; Riad et al., 2023).

The COVID-19 crisis and RUW, with their enormous impact on society, can be examined from the trauma perspective (Horesh and Brown, 2020; Jawaid et al., 2022). For individuals affected by the pandemic, this armed conflict has compounded an already traumatic situation, resulting in what is commonly referred to as double trauma (Brewin et al., 2000; Uwishema et al., 2022). The accumulation of these two traumas could have exacerbated their adverse effects on mental health and well-being, making it more challenging for individuals to recover and rebuild their lives (Jeftić et al., 2021; Chaaya et al., 2022; Kalaitzaki A. E. et al., 2022).

The detrimental effect of the RUW on mental health was investigated in civilian and military populations directly involved in the conflict, as well as in residents of other countries. A significant decline in global psychological well-being levels after the Russian invasion of Ukraine was observed among individuals across 17 European countries (Scharbert et al., 2024). Kurapov et al. (2022) and Xu et al. (2023) reported significant psychological distress among Ukrainian university students, personnel, and adults, with symptoms including depression, anxiety, emotional exhaustion, and insomnia, particularly during the early stages of the RUW. Increased stress, depression, and anxiety were also observed in those directly exposed to military actions (Kurapov et al., 2023). Similar clinical signs of mental health issues were found among Ukrainian military personnel and civilian defense volunteers (Pavlova et al., 2022). Overall, the war has significantly increased neurotic and stress-related disorders among Ukrainians (Yurtsenyuk and Sumariuk, 2023). Chudzicka-Czupała et al. (2023), who compared war-related mental health issues and coping strategies in populations from Ukraine, Poland, and Taiwan soon after the outbreak of the war, reported that Ukrainian participants showed significantly higher levels of anxiety, depression, and stress as compared to Polish and Taiwanese participants. More than half of the Polish and Taiwanese participants were distressed by the media war scenes. Polish university students who feared an armed attack by Russia on Poland reported significantly increased levels of anxiety than their less fearful peers (Skwirczyńska et al., 2022). Preoccupation with the RUW was related to higher levels of depression, anxiety, and stress in Italian adults, too (Barchielli et al., 2022). Raccanello et al. (2024) examined students’ coping and emotional responses from 16 countries during the second and third months of the RUW in 2022. The study revealed that anger and anxiety were the most common emotions, with hopelessness and hope also frequently reported. A clear pattern emerged, showing a greater prevalence of negative emotions over positive ones.

Although the Czech Republic is not a country directly involved in the ongoing RUW, the war has affected its population in many ways: by accepting a large number of refugees, providing significant financial and material aid to Ukraine, and facing its own economic insecurity, among other things. Remembering previous historical experience with the Russian invasion of Czechoslovakia in 1968, many people feared that the conflict could spread beyond Ukraine’s borders and directly affect the Czech Republic. The study of Czech university students by Riad et al. (2022) confirmed their deep concern about the RUW, with more than 35% of respondents reporting moderate to severe anxiety and 40% exhibiting moderate to severe depression.

Nonetheless, as Frankl (1992) has previously pointed out, each crisis or traumatic experience holds a “transformational potential,” suggesting that adversity and life-altering events can lead to positive psychological transformations. Tedeschi and Calhoun (2004, p. 1) introduced the term posttraumatic growth (PTG), which is defined as “the experience of positive change that occurs as a result of the struggle with highly challenging life circumstances.” The concept of PTG does not deny the harmful effects of traumatic events, nor does it aim to diminish empathy for the suffering of trauma survivors; instead, it offers a broader perspective on an individual’s life experiences (Linley and Joseph, 2011). Tedeschi and Calhoun (1995) distinguished three broad categories of posttraumatic growth: changes in self-perception, relationships, and spirituality. These are closely interconnected and permeate each other in certain respects. A later factor-analytic study yielded a five-factor conceptualization of PTG, which included domains of personal strength, new possibilities, relating to others, appreciation of life, and spiritual change (Tedeschi and Calhoun, 1996). More recently, a meta-analysis by Wu et al. (2019) found that nearly half of individuals who experienced a traumatic event reported moderate to high posttraumatic growth. Notably, higher rates were observed in those under 60 years of age, those working in specific professions, and those who experienced trauma directly.

Research indicates that the level of PTG achieved is related to the stress levels caused by the traumatic event (Wild and Paivio, 2003; Linley and Joseph, 2004; Tedeschi and Calhoun, 2006). Support for this comes from a comprehensive study by Matos et al. (2021), which surveyed more than 4,000 adults in 21 countries during the early stages of the COVID-19 pandemic. The findings suggest that PTG was more pronounced among individuals who perceived the pandemic as more stressful and demanding. Similar increases in PTG during the pandemic have been observed in other studies, particularly among those facing challenging life circumstances (e.g., Stallard et al., 2021; Yan et al., 2021; Collazo-Castiñeira et al., 2022; Ulset and von Soest, 2022). Kalaitzaki A. et al. (2022), who examined secondary traumatic stress and posttraumatic growth among Greek healthcare professionals during the COVID-19 lockdown, noted that PTG may co-occur with posttraumatic stress symptoms.

Posttraumatic growth as a positive outcome of distress has also been documented in refugees and populations affected by war (Ferriss and Forrest, 2018: Eltanamly et al., 2022; Stasielowicz, 2022). This may involve, in the first place, the development of a greater appreciation for life, increased resilience, and deepening personal relationships (Shakespeare-Finch and Lurie-Beck, 2014). In a longitudinal study on distress and PTG in Israeli ex-prisoners of war by Dekel et al. (2012), individuals with initial symptoms of posttraumatic stress disorder (PTSD) reported higher PTG levels over time than those without PTSD. PTG, on the other hand, had no effect on the subsequent development of PTSD symptoms. This adds further support to the idea that experiencing distress can facilitate and maintain growth.

Regarding the development of PTG directly related to RUW, Kokun (2023) found that in the initial months following the onset of the war in Ukraine, among the five domains of PTG, appreciation of life was ranked highest, succeeded by spiritual and existential shifts. Resilience, self-efficacy, professional commitment, control, and challenge acceptance were shown to be vital personal resources, fostering PTG in Ukrainian adults. Mottola et al. (2023) investigated the impact of the RUW on the mental health of Italians after 2 years of the pandemic. The study found that worrying about the war significantly increased stress and anxiety/depression. However, positive post-trauma changes and four aspects of growth (relating to others, new possibilities, personal strength, and spiritual change) reduced the impact of war concerns on anxiety and depression. Interestingly, a recent study of Syrian refugees showed that depression and anxiety symptoms might moderate the relationship between PTSD and PTG (Alpay, 2024). This might suggest that symptoms of psychological distress limit the degree to which PTG can be expected after a traumatizing experience. However, given the general nature of statistical interaction effects, the presented pattern of findings might have also resulted from a situation similar to the one described above, i.e., when PTG occurring in connection with the trauma leads to less severe PTSD-related symptoms of depression and anxiety. This interpretation is in line with the observation that although PTG and symptoms of psychological distress are both positively associated with traumatic experiences, the relationships between PTG and symptoms of anxiety and depression across a large number of studies have generally been weak to negligible (Long et al., 2021).

A key question for mental health promotion experts is what may increase the likelihood of posttraumatic growth in people exposed to a crisis such as war or a pandemic. In the military context, positive changes that indicate personal growth include both external and internal factors (Mark et al., 2018; Stein et al., 2018). Personality traits showing positive relationships with PTG include extraversion, agreeableness, openness to experience, and conscientiousness (Karanci et al., 2012; Mattson et al., 2018). In the setting of wartime conflicts, civilians’ ability to cope effectively with hardships has been associated with levels of self-efficacy (Ghosn et al., 2021), resilience (Mao and Agyapong, 2021), and hardiness (Diab et al., 2018).

Among the personal factors identified as enabling posttraumatic growth, maintaining a positive outlook on life plays a crucial role in successful coping with adversities. Optimistic and hopeful individuals may find it easier to identify and pursue new possibilities and strengths following trauma (Tedeschi and Calhoun, 1996; Snyder, 2000; Nolen-Hoeksema and Davis, 2002; Prati and Pietrantoni, 2009; Slezackova, 2017). Hope is critical in enabling people to perceive challenges instead of disasters and strategize their way through them. Snyder’s (2000) theory of hope defines it as a cognitive, motivational state that drives and sustains goal-oriented actions. According to this framework, dispositional hope is closely linked to individual goals and the requisite control over achieving these goals through personal effort. During the COVID-19 pandemic, higher dispositional hope was associated with greater well-being and lower perceived stress and anxiety levels among American adults (Gallagher et al., 2021).

On the other hand, a more recent concept of perceived hope represents a broader construct encompassing elements beyond personal control (Krafft et al., 2017). This form of hope involves a profound belief that things will unfold positively, independent of the results of personal endeavors. This broader understanding of hope can be more easily conceptually distinguished from optimism and future expectations, which often deal with more controllable outcomes (Montgomery et al., 2003; Krafft et al., 2017, 2021).

Research shows that hope is integral in dealing with life-threatening situations (Farran et al., 1995) and is a natural part of human existence, essential for living with future uncertainties (Nunn, 2005). Hope’s role extends to fostering a connection to something greater, such as peace, and enhancing personal growth by connecting to meaning in life (Peterson and Seligman, 2004; Cohn and Fredrickson, 2009; Krafft and Walker, 2018).

Numerous studies have explored the relationship between hope and PTG, revealing significant insights into how individuals navigate and grow from trauma. Hope has been identified as a protective factor against the harmful effects of trauma (Sympson, 2000; Scioli and Biller, 2009; Slezackova, 2017; Senger and Gallagher, 2024) and a crucial mediator between posttraumatic growth and self-efficacy (Chang and Kim, 2020). When combined with finding meaning after trauma, hope can lead to improved mental health outcomes (Ai et al., 2005). Predominantly studied within a clinical context, hope has been identified as a key factor in managing health challenges (Ho et al., 2011; Hullmann et al., 2014; Liu et al., 2020). Krafft et al. (2023) studied the role of hope in relation to well-being, coping styles, and PTG among adult respondents from 11 countries during the COVID-19 pandemic. Most of the participants reported moderate levels of posttraumatic growth, especially with regard to the appreciation of life and awareness of personal strengths. Furthermore, personal growth was positively associated with perceived hope and well-being. In the context of armed conflict, hope has been identified as an important predictor of societal resilience in the Czech population during the RUW (Koubová and Kimhi, 2024). Klicperova-Baker and Urban (2023) noted that feelings of hope related to the events in Ukraine and the situation in Czechia during the height of the poly-crisis in late 2022 were positively associated with the preference for democratic values. Kimhi et al. (2023) investigated the impact of the RUW on Ukrainian resilience and well-being five months after the invasion, comparing it with that of Israeli civilians during the May 2021 Israel-Gaza conflict. Ukrainian participants showed higher levels of distress and perceived threat but also reported greater hope and resilience — both societal and individual — than Israelis. According to Marciano et al. (2022), hope was found to be a strong predictor of coping with the COVID-19 pandemic and an armed conflict among Israeli adults. In Ukraine, hope, well-being, and morale were key predictors of resilience, outweighing the effects of perceived dangers and distress. The findings suggest that a war threatening national independence and sovereignty under certain conditions may boost societal resilience and hope among the affected population despite reduced well-being, increased distress, and perceived threats. An earlier study of soldiers who participated in the Ukrainian conflict after 2014 also showed that hope was strongly correlated to all dimensions of PTG (Kondratyuk and Puchalska-Wasyl, 2019).

**Figure 1:**
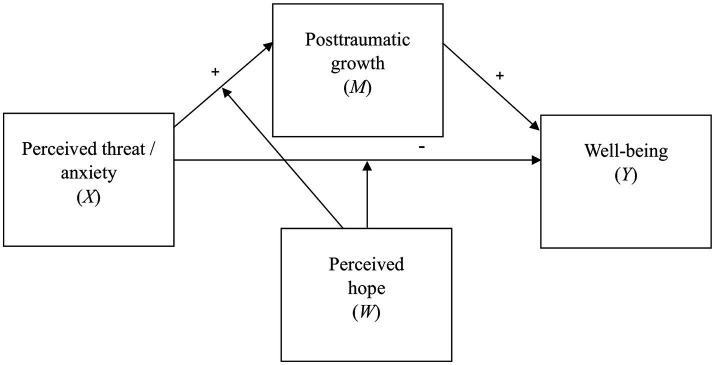
Conditional process model tested in the study.

The above findings illustrate the multifaceted relationship between hope, anxiety, PTG, and well-being. While previous research has mostly examined the relationship between hope and PTG separately from other factors, there is a need for further investigation into how hope may contribute to PTG in the context of the perceived threat of war. This study aims to fill this gap by exploring the role of hope in PTG among Czech adults whose lives have been affected by the Russian-Ukrainian War. Based on previous research on PTG, it can be expected that the level of PTG will determine whether perceived threat and the associated anxiety will or will not have a negative impact on well-being, i.e., that posttraumatic growth will moderate the relationships between perceived war threat and well-being as well as threat-related anxiety and well-being (Mottola et al., 2023). However, since posttraumatic growth itself is conceptualized as a potential outcome of stress associated with perceived threat, its additional conceptualization as a moderator of the effects of perceived threat and anxiety on well-being might be problematic (see Kraemer et al., 2002). Therefore, a more conceptually plausible model for our study would be one in which posttraumatic growth partially mediates the relationship between perceived threat or anxiety and well-being, and the degree to which perceived threat and anxiety are associated with posttraumatic growth is itself determined by one or more moderators. Furthermore, the direct and indirect effects of perceived threat and anxiety on well-being are expected to be in opposite directions (i.e., the effect mediated by posttraumatic growth should be positive rather than negative), which should be manifested as statistical suppression in a regression model in which posttraumatic growth is added to perceived threat or anxiety as a predictor of well-being. These relationships are together represented by the conditional process model (Hayes, 2022) displayed in Figure 1. In the present study, we specifically focused on perceived hope as a potential moderator of the relationships between perceived war threat and anxiety, and well-being. Our two main hypotheses were that perceived hope moderates the effects of both perceived war threat and anxiety on PTG. Specifically, we expected higher levels of hope to be associated with stronger positive effects of both predictors on PTG. In addition, we tested the hypothesis that hope also moderates the direct effects of both predictors on well-being, which would suggest that hope might mitigate the effects of distressing events on well-being even if other conditions of PTG are not met.

## 2 Method

### 2.1 Participants and data collection settings

The research sample was recruited by MNFORCE, a company that provides samples for online research based on a large national panel of participants. The sample composition aimed for the representativeness of the Czech Republic’s adult population aged 18 and over, with quota established for age, gender, region, urban/rural designation, and educational attainment. Participants agreed to the data collection company’s panel regulations and privacy policies and received compensation for their involvement. All participants were duly informed that the data collection served research purposes and provided consent to data processing by completing the questionnaire.

The sample consisted of 1,000 participants, 509 (50.9%) female and 491 male, between 23 and 65 years of age (M = 45.3, SD = 15.5). Regarding family status, 19% of respondents were single, 65% were married or living in a partnership, and 16% were divorced or widowed. Most respondents (57.1%) indicated full-time or part-time employment as their main activity; 24.2% identified as retired, 6.8% as students, 3.5 as unemployed, and 8.4 as staying at home for parenting/family reasons. Regarding achieved education level, 14% of the participants were college graduates, 34.7% had completed secondary education with a school-leaving certificate, 46.8% had completed secondary education without a certificate, and 4.4% had only completed primary education.

### 2.2 Instruments

*The Perceived Hope Scale* (PHS; Krafft et al., 2017) was designed as a unidimensional measure of hope as directly perceived by people, conceptually distinct from the construct of agency and optimism, which are more closely linked to future expectancies of goal attainment (Krafft et al., 2021; Marujo et al., 2021). The six items, rated on a Likert scale ranging from 1 (“Strongly disagree”) to 6 (“Strongly agree”), ask about the respondent’s general level of hope, their broad belief in the fulfillment of their hopes, how far their hope outweighs anxiety, whether hope improves their quality of life, and whether they are able to remain hopeful in difficult times. The Czech version of the scale has exhibited good levels of construct validity and internal consistency (Slezackova et al., 2020).

*General Anxiety Disorder* (GAD-7; Spitzer et al., 2006) is an instrument for the assessment of general symptoms of anxiety. Its seven items are rated on a scale from 1 (“Not at all”) to 4 (“Almost every day”).

*The Posttraumatic Growth Inventory* (PTGI – SF; Cann et al., 2010) is a 10-item measure of PTG, capturing all of the five factors of the original 21-item scale (Tedeschi and Calhoun, 1996): appreciation of life, relating to others, personal strength, new possibilities, and spiritual change. Each factor is represented by two items. In the present study, respondents used a 6-point scale to indicate the extent to which there was a positive change in their lives in 2022 as a result of the situation associated with the war in Ukraine (1 = “This change did not occur at all,” 6 = “This change occurred to a great extent”). Following Cann et al. (2010), the total PTG score, rather than the subscores, was used in our data analyses.

*The Mental Health Continuum – Short Form* (MHC-SF; Keyes, 2005; Czech version by Singh et al., 2016) is a measure of the hedonic, psychological, and social aspects of well-being. It consists of 14 items rated on a 6-point Likert scale indicating how often the respondent experiences a certain situation or state (1 = “Never “, 6 = “Every day”). The hedonic well-being subscale, consisting of 3 items, is a measure of subjective emotional experience. The psychological well-being subscale, consisting of 6 items, is a measure of subjective perception of one’s own adaptive functioning on the personal level. Finally, the social well-being subscale, comprising 5 items, measures subjective perception of one’s functioning in society and the functioning of society in general.

*Perceived threat of the Russian-Ukrainian war* was assessed by a single item, “I feel threatened by the war in Ukraine,” rated on a scale from 1 (“Strongly disagree”) to 5 (“Strongly agree”).

Each of the above measures, except the last one, was scored by averaging across the respective item scores. Results of the reliability analysis of all these instruments are available in Table 1.

**Table 1:** Descriptive statistics and internal consistencies of self-report measures in the study.

	*M*	SD	Skewness	McDonald’s ω total /Pearson r^*^
PTGI-SF				
Appreciation of life	3.07	1.52	0.22	0.68^*^
New possibilities	2.48	1.36	0.71	0.67^*^
Spirituality	2.05	1.34	1.21	0.78^*^
Relationships	2.61	1.32	0.53	0.60^*^
Strength	2.69	1.44	0.44	0.79^*^
Total posttraumatic growth	2.60	1.19	0.49	0.93
Anxiety (GAD-7)	2.02	0.71	0.62	0.92
Perceived threat of war	3.19	1.26	−0.26	–
Perceived hope (PHS)	3.79	1.06	−0.43	0.94
MHC				
Hedonic	3.99	1.33	−0.43	0.88
Psychological	3.68	1.17	−0.28	0.87
Social	2.72	1.17	0.69	0.83
Total Well-being	3.40	1.08	−0.06	0.92

^*^Reported for two-item measures.

### 2.3 Data analysis

Because the psychometric properties of the Czech translation of PTGI-SF have not been previously tested, we conducted a confirmatory factor analysis prior to proceeding with the main data analysis to verify the five-dimensional structure of the measure as well as to determine whether the use of a total score was justified. The analysis was performed in R using the lavaan package (Rosseel, 2012). The models were estimated using the Satorra-Bentler robust maximum likelihood estimation (Satorra and Bentler, 1994), which has been shown to perform at a satisfactory level with Likert-type scales with 5 points or more, even when the assumption of normality is not met (Bovaird and Koziol, 2012). We tested a hierarchical model with each of the five original dimensions of PTGI-SF loading on a single higher-order factor, and this model was compared with a more parsimonious unidimensional model based on model fit, which was assessed using the robust Root Mean Square Error of Approximation (RMSEA) with 90% CIs (acceptable fit: UCI < 0.10), robust standardized root mean square residual (acceptable fit: SRMR <0.08; Hu and Bentler, 1999), and robust Comparative Fit Index (CFI) and Tucker Lewis Index (TLI), both with a cutoff criterion of 0.90 for minimum acceptable fit, and values of 0.95 and above considered as good fit (Hu and Bentler, 1999).

All subsequent analyses were conducted in IBM SPSS Statistics, version 29. First, descriptive statistics and Pearson correlations between all included measures were computed, and internal consistencies of all multi-item measures were assessed using McDonald’s omega.

The moderation effects were tested and explored using the PROCESS macro for SPSS, version 4.2, introduced by Hayes (2022). Predictor variables were mean-centered prior to the analysis. Standardized coefficients were obtained by re-running the moderation analyses with standardized predictors, moderators, and outcomes (see Hayes, 2022). The Johnson-Neyman probing technique was employed to determine the boundaries of significance for the conditioned effects. The nature of the moderation effect was explored using line graphs depicting the effects of the predictor on the outcome at the 16th, 50th, and 84th percentile of the moderator variable (see Hayes, 2022). Confidence intervals for determining significance were estimated using bootstrapping with 5,000 samples. Subsequently, conditional process analysis (Hayes, 2022) was conducted using the PROCESS macro to test the overall conditional process model depicted in Figure 1.

Mathematically, both models are represented by regression Eqs. 1, 2, where *X* represents the predictor (perceived threat of war or anxiety), *M* represents posttraumatic growth as the mediator variable, W represents perceived hope as the moderator variable, *Y* represents subjective well-being as the outcome, a_0_ and b_0_ represent model constants, a_1_, a_2_, a_3_, b_1_, c_1_, c_2_ and c_3_ represent the estimated effects of predictor terms, and *e_M_* and *e_Y_* represent prediction errors.


(1) $$M=a_{0}+a_{1}X+a_{2}W+a_{3}XW+e_{M}$$

(2) $$Y=b_{0}+b_{1}M+c_{1}X+c_{2}W+cXW+e_{Y}$$

The significance of the conditioned indirect effects of the predictor variables on well-being through posttraumatic growth was tested using the percentile bootstrap confidence interval (CI) method with 5,000 bootstrap samples.

Before interpreting the results of the regression analyses, potential violations of the assumption of homoscedasticity were evaluated by inspecting residual plots for each model tested. These did not seem to indicate heteroscedasticity in any of the models. In addition, independence of errors was tested using the Durbin-Watson statistic (with values ranging between 0 and 4, and a value of 2 indicating zero correlation between residuals; Durbin and Watson, 1951). Values of the Durbin-Watson statistic varied between 1.83 and 2.06, indicating no substantial correlations between errors across the models tested.

## 3 Results

### 3.1 Confirmatory factor analysis of PTGI-SF

The scale’s proposed five-factor structure and the adequacy of computing a total posttraumatic growth score were evaluated using confirmatory factor analysis. The target model we tested was a hierarchical model with the five dimensions loading on a single second-order factor. The model was identified after fixing one of the second-order loadings to 1. The model showed a good fit (χ^2^(30) = 155.57, *p* < 0.001; CFI = 0.97; TLI = 0.95; RMSEA = 0.09 [0.07, 0.10]; SRMR = 0.04), superior to the fit of the unidimensional model, in which all items loaded directly on a single factor (χ^2^(34) = 243.04, *p* < 0.001; CFI = 0.95; TLI = 0.93; RMSEA = 0.11 [0.09, 0.12]; SRMR = 0.05). The sizes of the first-order loadings ranged from 0.72 to 0.95. The second-order loadings of the factors of Appreciation of Life, New Possibilities, Relationships, and Strength on the second-order factors were all above 0.90. The loading of the Spirituality factor on the second-order factor was somewhat lower (0.69).

Overall, it was concluded the measure showed acceptable construct validity, and the subsequent analyses were conducted with the total PTGI-SF score (“posttraumatic growth”; PTG).

### 3.2 Descriptive statistics

Descriptive statistics of all measures are summarized in Table 1. For each PTGI-SF subscale and the total posttraumatic growth score, values could range from 0 to 5. The generally positive skew indicates that most people reported relatively low levels of posttraumatic growth, especially in the spiritual domain. The highest levels of posttraumatic growth were reported with respect to appreciation of life. Most people also reported relatively low levels of anxiety (with a theoretical range of GAD-7 scores between 1 and 4) but a relatively high perceived personal threat of war in Ukraine, with 47.8% expressing agreement or strong agreement – as opposed to 32.4% expressing disagreement or strong disagreement – with the statement “I feel threatened by the war in Ukraine.” Reported levels of perceived hope and total well-being were moderate. Hedonic and psychological well-being levels were generally higher than social well-being levels.

### 3.3 Correlation analysis

Table 2 shows correlations between all of the five measures included in further analyses. The results of the Pearson correlation analysis indicated, as expected, a strong positive relationship between hope and overall well-being, and negative correlations between anxiety and both perceived hope and well-being, which were somewhat weaker in comparison. Perceived threat of war was weakly positively related to anxiety, while its relationships with perceived hope and well-being were close to zero. Consistent with the expectations, posttraumatic growth was weakly positively associated with all measures. These patterns of relationships generally correspond to those one would expect if the proposed moderation effects were at play.

**Table 2:** Pearson correlations between all self-report measures in the study.

	1.	2.	3.	4.
1.Posttraumatic growth	–			
2.Anxiety	0.15^**^	–		
3.Perceived threat of war	0.18^**^	0.26^**^	–	
4.Perceived hope	0.24^**^	−0.32^**^	0.04	–
5.Well-being	0.28^**^	−0.27^**^	−0.00	0.58^**^

^*^p < 0.05; ^**^p < 0.001.Derived from bootstrap confidence intervals based on 10,000 samples.

### 3.4 Moderation of the effects of perceived threat of war and anxiety on posttraumatic growth by perceived hope

For the sake of clarity, testing of the conditional process models represented by Figure 1 was broken down into two steps. In the first step, we tested the moderation effects of perceived hope separately, i.e., we first tested models represented by Eq. 1.

**Figure 2:**
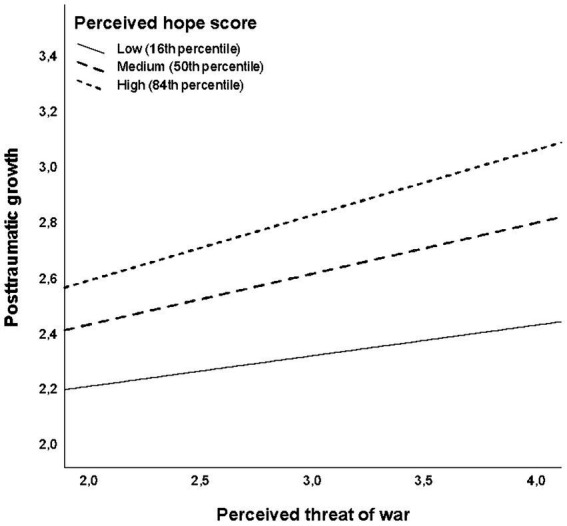
The effect of the perceived threat of war on posttraumatic growth as moderated by perceived hope.

Table 3 shows the results of the first moderation analysis, in which perceived hope was tested as a moderator of the relationship between the perceived threat of war and posttraumatic growth. A model testing the unconditional effects of the perceived threat of war and perceived hope (Model 1 in Table 3) confirmed both variables as significant albeit weak positive predictors of posttraumatic growth (*F*(2, 997) = 46.92, *p* < 0.001, *R* = 0.29). Introducing an interaction term (Model 2 in Table 3) added 0.6% of explained variance, and this increment proved significant (*F_change_*(1, 996) = 6.83, *p* = 0.009; Model fit: *F*(3, 996) = 33.74, *p* < 0.001, *R* = 0.30). The graph in Figure 2 indicates that this weak but significant moderation effect of perceived hope was in the expected direction, which means that the perceived threat of war was a stronger positive predictor of posttraumatic growth in participants scoring higher on perceived hope than in those scoring lower. Subsequent Johnson-Neyman probing technique helped to determine the perceived hope cut-off score of 2.40 as the point below which the positive relationship between the perceived threat of war and posttraumatic growth was no longer significant. At the minimum perceived hope score of 1, the predicted effect of the perceived threat of war on posttraumatic growth was close to zero.

**Table 3:** Multiple regression of posttraumatic growth with perceived threat of war as a predictor and perceived hope as a moderator.

		*B*	SE_B_	*β*	*p*	*_Δ_R^2^*	*p*_change_
Model 1	Perceived threat of war	**0.16**	**0.03**	**0.17**	**< 0.01**	0.09	< 0.01
	Perceived hope	**0.26**	**0.03**	**0.22**	**< 0.01**		
Model 2	Perceived threat of war	**0.17**	**0.03**	**0.18**	**< 0.01**	0.01	< 0.01
	Perceived hope	**0.26**	**0.03**	**0.24**	**< 0.01**		
	PTW × PH	**0.06**	**0.02**	**0.07**	**< 0.01**		

Both predictors were mean-centered before running the analysis.

**Figure 3:**
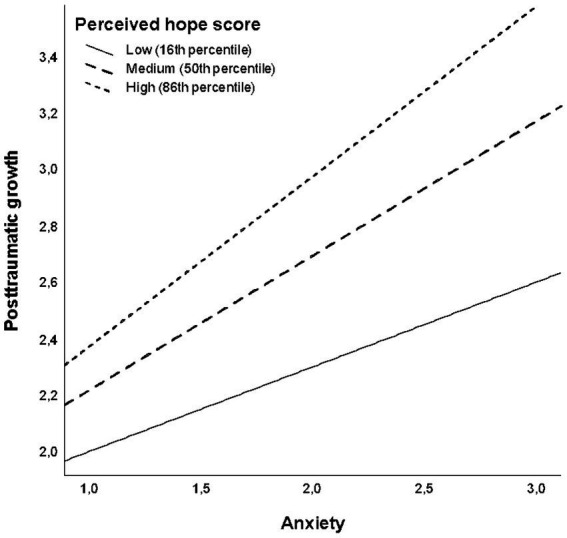
The effect of anxiety on posttraumatic growth as moderated by perceived hope.

The moderation effect of perceived hope on the relationships between anxiety and posttraumatic growth (Table 4) was stronger compared to the previous model. In the model without the interaction term, anxiety and perceived hope were both significant positive predictors of posttraumatic growth (see Model 1 in Table 4; *F*(2, 997) = 64.36, *p* < 0.001, *R* = 0.34). Adding anxiety × perceived hope interaction (Model 2 in Table 4) significantly increased explained variance by 1.2% (*F_change_*(1, 996) = 13.74, *p* < 0.001; Model fit: *F*(3, 996) = 48.03, *p* < 0.001, *R* = 0.36). The nature of the interaction effect is depicted in Figure 3, which – as with perceived threat of war – indicates that the strength of the positive relationship between anxiety and posttraumatic growth increased with an increasing level of perceived hope. The Johnson-Neyman probing technique identified the perceived hope score of 1.98 as the cut-off point below which the relationship between anxiety and posttraumatic growth was no longer significant. Similar to the previous model, the predicted effect of the perceived threat of war on posttraumatic growth at the minimum level of perceived hope was close to zero.

**Table 4:** Multiple regression of posttraumatic growth with anxiety as a predictor and perceived hope as a moderator.

		*B*	SE_B_	*β*	*p*	*_Δ_R^2^*	*p*_change_
Model 1	Anxiety	**0.43**	**0.05**	**0.26**	**< 0.01**	0.11	< 0.01
	Perceived hope	**0.35**	**0.04**	**0.32**	**< 0.01**		
Model 2	Anxiety	**0.45**	**0.05**	**0.27**	**< 0.01**	0.01	< 0.01
	Perceived hope	**0.34**	**0.04**	**0.31**	**< 0.01**		
	Anxiety × PH	**0.15**	**0.04**	**0.10**	**< 0.01**		

Both predictors were mean-centered before running the analysis.

### 3.5 The conditional direct and indirect effects of perceived threat of war and anxiety on well-being with posttraumatic growth as a mediator

Having established the hypothesized moderating effects of perceived hope on the relationships between perceived threat of war and posttraumatic growth as well as anxiety and posttraumatic growth, we proceeded with testing the models represented by Eq. 2. The model in which mental health was predicted by posttraumatic growth and perceived threat of war, and the effect of perceived threat of war was moderated by perceived hope, is summarized in Table 5 (Model 1; *F*(4, 995) = 143.27, *p* < 0.001, *R* = 0.60). Since the moderation effect of perceived hope was very weak and non-significant, we repeated the analysis without the interaction term (Model 2; *F*(3, 996) = 190.24, *p* < 0.001, *R* = 0.60). It turned out that perceived hope was a much stronger predictor of mental health than either posttraumatic growth, which was a weak significant positive predictor of mental health, or perceived threat of war, which was a very weak but still significant negative predictor of mental health. After removing perceived hope from the equation (Model 3; *F*(2, 997) = 42.58, *p* < 0.001, *R* = 0.28), the effect of posttraumatic growth slightly increased.

**Table 5:** Multiple regression of mental health with perceived threat of war and posttraumatic growth as predictors and perceived hope as a moderator (see Eq. 2).

		*B*	SE_B_	β	*p*	*R^2^*
Model 1	Posttraumatic growth	**0.14**	**0.02**	**0.16**	**< 0.01**	**0.37**
	Perceived threat of war	**−0.05**	**0.02**	**−0.06**	**0.03**	
	Perceived hope	**0.55**	**0.03**	**0.55**	**< 0.01**	
	PTW × PH	−0.03	0.02	−0.03	0.17	
Model 2	Posttraumatic growth	**0.14**	**0.02**	**0.16**	**< 0.01**	**0.36**
	Perceived threat of war	**−0.04**	**0.02**	**−0.05**	**0.04**	
	Perceived hope	**0.56**	**0.03**	**0.55**	**< 0.01**	
Model 3	Posttraumatic growth	**0.26**	**0.03**	**0.29**	**< 0.01**	**0.08**
	Perceived threat of war	−0.05	0.03	−0.05	0.08	

Both predictors were mean-centered before running the analysis.

A similar situation was observed in the model with anxiety (Table 6). Again, the moderation effect of perceived hope was weak and non-significant (Model 1; *F*(4, 995) = 152.38, *p* < 0.001, *R* = 0.62). In the linear regression model without the interaction term (Model 2; *F*(3, 996) = 202.20, *p* < 0.001, *R* = 0.62), posttraumatic growth was a significant positive predictor and anxiety a significant negative predictor of mental health, above and beyond perceived hope, which was strongly positively associated with mental health. Both effects increased when perceived hope was removed from the regression equation (Model 3; *F*(2, 997) = 108.35, *p* < 0.001, *R* = 0.42).

**Table 6:** Multiple regression of mental health with anxiety and posttraumatic growth as predictors and perceived hope as a moderator (see Eq. 2).

		*B*	SE_B_	β	*p*	*R^2^*
Model 1	Posttraumatic growth	**0.17**	**0.02**	**0.18**	**< 0.01**	**0.38**
	Anxiety	**−0.22**	**0.04**	**−0.15**	**< 0.01**	
	Perceived hope	**0.50**	**0.03**	**0.50**	**< 0.01**	
	Anxiety × PH	−0.05	0.03	−0.03	0.14	
Model 2	Posttraumatic growth	**0.16**	**0.02**	**0.18**	**< 0.01**	**0.38**
	Anxiety	**−0.21**	**0.04**	**−0.14**	**< 0.01**	
	Perceived hope	**0.50**	**0.03**	**0.50**	**< 0.01**	
Model 3	Posttraumatic growth	**0.29**	**0.03**	**0.33**	**< 0.01**	**0.18**
	Anxiety	**−0.49**	**0.04**	**−0.32**	**< 0.01**	

Both predictors were mean-centered before running the analysis.

**Figure 4:**
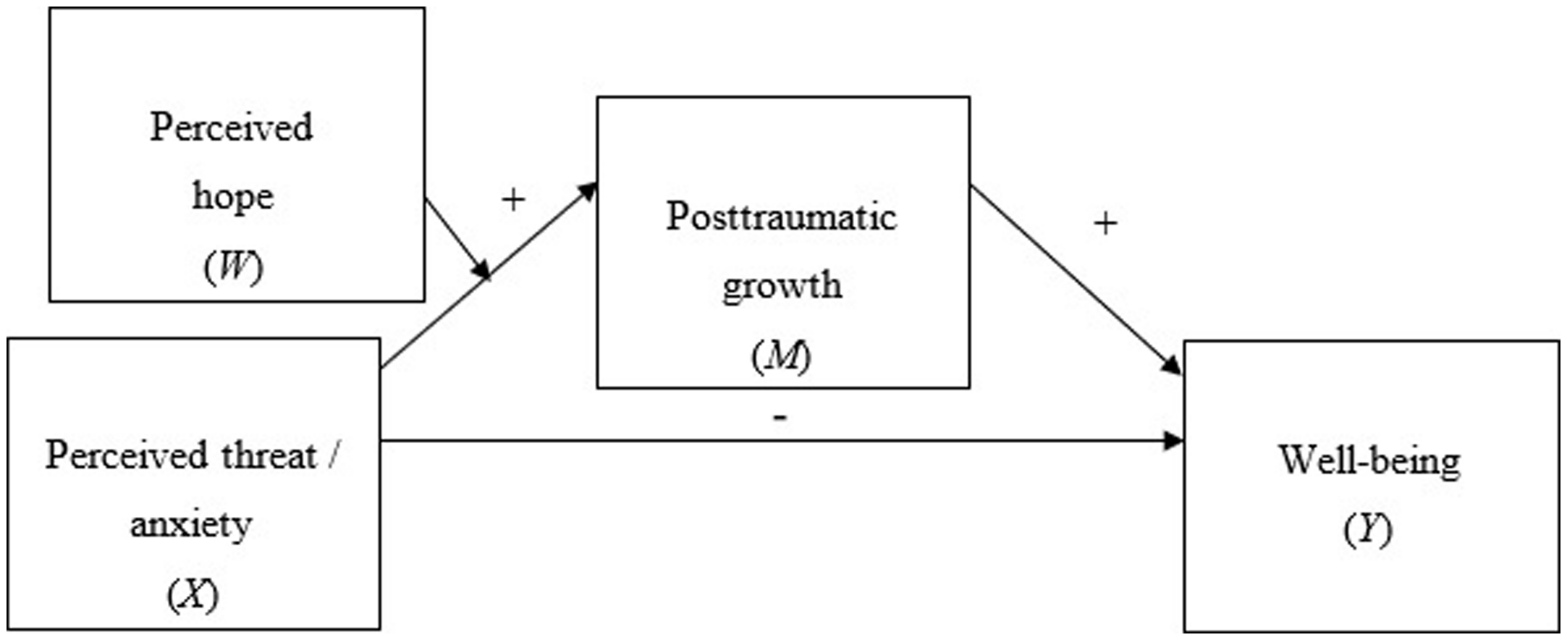
Final conditional process model supported by the study data.

The last step in the conditional process analysis was to estimate the sizes of the conditional indirect and direct effects of both predictors on the outcome. However, since a significant moderation of the direct effect was not established for either of the two original conditional process models, we re-ran the analyses with simplified models, represented by Figure 4.

In this model, perceived hope still moderates the effect of the predictor (either perceived threat of war or anxiety) on the mediator (posttraumatic growth). However, it does not moderate the direct effect of the predictor on the outcome (mental health), which means that only the indirect effect of the predictor on the outcome is conditional. The simplified model is represented by Eqs.3, 4.


(3) $$M=a_{0}+a_{1}X+a_{2}W+a_{3}XW+e_{M}$$

(4) $$Y=b_{0}+b_{1}M+b_{2}X+e_{Y}$$

A summary of the conditional indirect effects of both perceived threat of war and anxiety on mental health through posttraumatic growth is provided in Table 7. The results show that the indirect effect’s size increased with an increasing level of the moderator in both models. This means that, in individuals with higher levels of perceived hope, both perceived threat of war and anxiety had somewhat stronger positive effects on mental health through posttraumatic growth. The effects of the moderator on both indirect effects were significant (the index of moderated mediation with bootstrapped 95% CI was 0.02 [0.00, 0.03] for perceived threat of war and 0.04 [0.02, 0.07] for anxiety). However, it must be noted that, albeit weak, the positive indirect effects of predictors on mental health through posttraumatic growth were significant even at relatively low levels of perceived hope. Interestingly, in the model with perceived threat of war, the direct effect of the predictor on mental health was not significant (*B* = −0.05, *p* = 0.08; see Model 3 in Table 5). In contrast, in the model with anxiety, the direct negative effect of the predictor on mental health was substantially stronger than its indirect effect through posttraumatic growth across different values of the moderator (*B* = −0.49, *p* < 0.001, see Model 3 in Table 6).

**Table 7:** Conditional indirect effects of perceived threat of war (Model 1) and anxiety (Model 2) on mental health through posttraumatic growth at different levels of perceived hope.

Predictor	Perceived hope	Unstand. estimate	95% bootstrap CI	Stand. estimate
Perceived threat of war (Model 1)	Low (16th percentile)	**0.03**	**0.01, 0.05**	**0.03**
	Medium (50th percentile)	**0.05**	**0.03, 0.07**	**0.06**
	High (84th percentile)	**0.06**	**0.04, 0.09**	**0.07**
Anxiety (Model 2)	Low (16th percentile)	**0.09**	**0.05, 0.13**	**0.06**
	Medium (50th percentile)	**0.14**	**0.10, 0.19**	**0.09**
	High (84th percentile)	**0.18**	**0.12, 0.24**	**0.12**

Both predictors were mean-centered before running the analysis.

## 4 Discussion

The present study investigated the role of perceived hope in the development of posttraumatic growth in response to perceived personal threat and anxiety associated with the war in Ukraine in the Czech adult population. In addition, we examined the relationships of all of these variables with mental well-being. Specifically, we tested two conditional process models, one for perceived personal threat of war and one for anxiety, in which one of the two variables predicted well-being as measured by the Mental Health Continuum (i.e., hedonic, psychological, and social well-being) both directly and indirectly through posttraumatic growth. Both perceived threat of war and anxiety were expected to have a negative direct effect on well-being. At the same time, however, they were expected to have a positive indirect effect on well-being through posttraumatic growth. Conceptually, posttraumatic growth is expected to arise from stressful experiences, which are characterized by perceived threat and anxiety, when certain conditions are met. Therefore, we focused on perceived hope as a potential moderator of the relationships between perceived threat of war and posttraumatic growth and between anxiety and posttraumatic growth. Specifically, we expected stronger and positive effects of perceived threat of war and anxiety on PTG in people with high levels of perceived hope compared to people with low levels of perceived hope. Simultaneously, we tested the hypothesis that perceived hope also moderates the direct negative effects of perceived threat and anxiety on well-being, i.e., that perceived hope might act as a protective factor against the detrimental effects of stress on well-being even if the stress does not result in posttraumatic growth.

Our analysis revealed some striking aspects of the psychological responses to the war threat among the Czech population. In our sample, posttraumatic growth levels were generally low, particularly in the spiritual domain. In line with previous studies (Shakespeare-Finch and Lurie-Beck, 2014; Kokun, 2023; Krafft et al., 2023), the highest levels of PTG were observed in the domain of appreciation of life. Furthermore, while most participants reported relatively low anxiety levels, they also indicated a relatively high perceived personal threat from the war in Ukraine. This finding is consistent with the outcomes of other studies, pointing at increased levels of fear and preoccupation with the RUW in Polish and Italian samples (Barchielli et al., 2022; Skwirczyńska et al., 2022). In line with the outcomes of the recent reviews (Lee and Gallagher, 2018; Pleeging et al., 2021; Murphy, 2023), we also found a strong positive correlation between hope and mental well-being. Unsurprisingly, we were also able to replicate the previously observed negative correlations between anxiety and both hope (Barlow, 2000; Gallagher et al., 2021; Richardson, 2023) and well-being (Kinderman et al., 2015). Perceived threat of war showed a slight positive correlation with anxiety, which is indicative of a natural human emotional response to perceived endangerment. However, its links to perceived hope and well-being were close to zero. Therefore, we tried to shed more light on these mutual relationships in follow-up analyses, the results of which are discussed below. Posttraumatic growth showed weak positive associations with all variables measured, which supports the findings of previous works pointing at the associations between PTG and well-being (Chen et al., 2022; Munroe and Ferrari, 2022; Pięta and Rzeszutek, 2022), hope (Scioli and Biller, 2009; Slezackova, 2017; Kondratyuk and Puchalska-Wasyl, 2019; Krafft et al., 2023), and symptoms of distress (Satheesan and Hameed, 2017; Mottola et al., 2023).

Our study also revealed some limitations in the role of perceived hope. The results of the analyses do not support the direct effect of the perceived threat of war on well-being or the direct effect of anxiety on well-being. In other words, perceived hope did not significantly alter the effects of perceived threat and anxiety on well-being independently of posttraumatic growth.

However, we did find evidence for the moderating role of perceived hope in the effects of both perceived threat of war and anxiety on PTG. Significant indexes of moderated mediation in the full conditional process model indicated that perceived hope not only significantly moderated the effects of perceived threat of war and anxiety on posttraumatic growth but also affected the strength of the entire indirect path between perceived threat of war and well-being and that between anxiety and well-being: The positive effect of both stress-related predictors on well-being through posttraumatic growth was stronger at higher levels of perceived hope as opposed to low levels of perceived hope, although in both models the indirect effect remained significant (albeit weak) even at the lower 16th percentile of perceived hope scores. In a model assuming causality, this would mean that high perceived hope provides a condition for posttraumatic growth to occur under stress associated with the threat of war; however, it does not seem to protect from the detrimental effects of perceived threat on well-being by simply dampening the negative emotional reaction associated with the threat. At very low levels of perceived hope, neither perceived threat of war nor anxiety had a significant effect on posttraumatic growth. However, at moderate to high levels of perceived hope, both the perceived threat of war and anxiety had positive effects on posttraumatic growth. In other words, perceived threat and anxiety may lead to the development of PTG, which, in turn, is positively related to well-being. Whether perceived threat and/or anxiety will result in PTG appears to be partly conditional on the level of perceived hope, with more hopeful individuals being more likely to experience posttraumatic growth.

Our results indicate that while hope can enhance growth and adaptation in the face of traumatogenic event, it may not suffice as a standalone buffer against the more immediate psychological impacts of stress and anxiety. Nevertheless, our findings underscore the role of hope in traumatic contexts, similar to the transformational potential of adversity suggested by Frankl (1992) and Tedeschi and Calhoun (2004). In the context of dual trauma such as that experienced during the COVID-19 pandemic followed by the Russia-Ukraine war, hope can play a crucial role in enabling PTG, even as direct emotional distress persists. Similar to studies conducted during the COVID-19 pandemic (Matos et al., 2021) and other traumatic contexts (Eltanamly et al., 2022), our results might suggest that hope can act as a facilitator for PTG amidst adversity, enhancing individuals’ capacity to derive growth from the challenges of war. This is in line with the idea that while direct mitigation of stress effects on well-being may require additional factors, hope plays a pivotal role in leveraging stressful experiences toward personal and psychological growth. Our findings align with and extend the observations made by Barchielli et al. (2022) and Riad et al. (2022), which highlighted the pervasive psychological impact of the Russia-Ukraine war across different populations. Unlike these studies, which emphasize direct emotional impacts, our research points to the nuanced role of hope in fostering growth beyond immediate distress, echoing Kalaitzaki A. et al. (2022)‘s observation that PTG can coexist with posttraumatic stress symptoms. This duality suggests that immediate emotional responses to trauma are natural but not definitive of one’s complete psychological state. The process of awareness of hope modulates how we interpret the stress of PTG and modulates our well-being. On the other hand, our findings diverge from studies like those by Kurapov et al. (2022) and Raccanello et al. (2024), which highlighted the significant direct impacts of war-related stress on mental health. In contrast to these studies, our research suggests that the positive transformative effects of stress, facilitated by hope, are more pronounced in PTG rather than direct enhancement of well-being. This divergence may be due to cultural or contextual differences in the experience and utilization of hope and resilience.

There are some additional findings that require attention, providing context to the results above. First, it should be noted that while the average score of perceived threat of war in our sample was relatively high (i.e., above the scale midpoint of 3.0), the first-order correlation between this variable and anxiety was rather weak (*r* = 0.26), and – even more surprisingly – there was a near-zero correlation between perceived threat of war and well-being as measured by the Mental Health Continuum. Indeed, the association between perceived threat of war and PTG (*r* = 0.18) was stronger in our sample than between perceived threat of war and well-being, and it was confirmed by the conditional process analysis that perceived threat of war was only significantly associated with mental health indirectly and positively (albeit weakly) through posttraumatic growth. The expected significant direct negative effect was not found. With anxiety, on the other hand, the expected significant direct effect on well-being was observed and was substantially stronger than the indirect effect. This suggests that perceived threat, while recognized, does not necessarily translate into direct emotional distress or reduced mental health as might be expected. Instead, the stronger correlation with posttraumatic growth – as compared to that with well-being – suggests that the perception of threat may activate a more reflective or meaning-making response among individuals rather than inducing a direct stress response. These results further support the observation that the impact of perceived war threat on well-being is mediated by posttraumatic growth, albeit weakly (Mottola et al., 2023). In other words, this indicates that even in the absence of a strong direct impact on mental health, the experience of threat can still contribute positively to psychological development through mechanisms of growth and adaptation. The significant direct effect of anxiety on well-being, in contrast, underscores the more typical response expected from a stressor, where increased anxiety clearly diminishes mental health.

The second observation relevant to the present findings is that the levels of posttraumatic growth were generally low (*M* = 2.6, which can be compared to the scale midpoint of 3.5), with the highest scores observed with the subscale Appreciation of Life. One possible interpretation of this is that although the Ukrainian war triggered some awareness of potential personal impact in adult Czechs, it did not necessarily lead to a fully developed posttraumatic stress response but, in more hopeful individuals, was more likely to result in reflecting on their own blessings in the shadow of personal tragedies witnessed in a fellow country plagued by war. This reflects a kind of selective engagement with the crisis, where direct threats do not lead to a complete stress response but encourage a reassessment of personal values and blessings, which can be intricately linked to hope, gratitude, and spirituality. This is consistent with literature suggesting that hope is closely associated with gratitude (McCullough, 2002; Witvliet et al., 2018; Bazargan-Hejazi et al., 2023) - a recognition and thankful appreciation of what one has, which can be particularly poignant in times of broader societal suffering (e.g., during an ongoing war in the neighboring countries). Hope also often intersects with the meaningfulness of life (Hammer et al., 2009; Feldman et al., 2018; Guse and Shaw, 2018; Karaman et al., 2020), which provides a framework for understanding one’s place in the world and the meaning behind events, even traumatic ones (Ai et al., 2005; Linley and Joseph, 2011). People who recognize meaning in life are fundamentally more hopeful, significantly reducing the negative psychological consequences after traumatic experiences (Krafft and Walker, 2018).

Overall, our research suggests that in the context of high perceived threat and anxiety, such as those arising from the RUW, individuals with higher levels of perceived hope are more likely to experience PTG. This aligns with the theoretical premise that hopeful individuals exhibit resilience and positively reevaluate stressful events. Importantly, our results highlight that while perceived hope does not significantly modulate the direct effects of perceived threats and anxiety on well-being, it does enhance the positive relationship between these stressors and PTG, especially at moderate to high levels of hope.

To summarize the above findings, we can apply a metaphor of hope as a catalyst in a chemical reaction. In the context of war-induced stress, hope does not prevent the initial emotional strain — people still feel the impact of threats and anxiety. However, hope activates a transformative process, converting these negative experiences into growth opportunities. This process appears to be most effective when hope levels are at least moderate, suggesting that a certain threshold of hope is necessary to kickstart the growth process.

These insights have significant implications for developing interventions aimed at supporting psychological well-being and fostering posttraumatic growth (PTG) among individuals affected by war and other traumatic events, particularly those experiencing high levels of perceived threat and anxiety. Integrating programs that build and sustain hope into broader psychological support services can be highly beneficial for individuals in conflict zones or those recovering from traumatic events. Therapeutic practices can incorporate techniques such as identifying and nurturing sources of hope, finding meaning, and cultivating a positive outlook on life to help individuals maintain a hopeful perspective despite challenging circumstances. Community-based interventions that foster collective hope through initiatives promoting community cohesion and mutual support can further enhance the overall psychological resilience of the community. These strategies, focused on both individual and collective hope, can significantly contribute to the recovery and growth of trauma-affected populations.

### 4.1 Limitations and future directions

This study brings novel findings about the psychological impact of war in Ukraine on a large sample of Czech adults and underscores the complexity of relationships between threat perception, emotional responses, well-being, and posttraumatic growth. Although we see our findings as valuable and relevant, the study has some limitations to be pointed out. One of them is its reliance on self-reported measures and a single cultural context, which might not capture the whole variety of responses to RUW-related trauma seen in more diverse populations.

Furthermore, the cross-sectional design of our study does not allow direct assessment of causality, which has important implications for the interpretations of our findings. Namely, although the models presented in our study seem particularly theoretically plausible, the observed pattern of statistical associations could also result from other patterns of underlying causal relations. For example, the experience of hope might be the result - rather than a cause - of the connection between perceived threat or anxiety and posttraumatic growth. In such a model, however, the conditions under which posttraumatic growth occurs would still have to be specified.

Another potential limitation in our study is our reliance on a single-item measure for the measurement of perceived threat of war. A more comprehensive measure might capture the construct more fully and reduce the risk of different participants understanding the broadly formulated question in different ways.

Future research incorporating longitudinal designs should investigate how perceived hope evolves over time in individuals affected by war and other traumatic events. Understanding these temporal dynamics can provide insights into the stability of hope and its long-term impact on PTG, including whether initial levels of hope predict sustained growth or if fluctuations in hope correlate with changes in PTG. Additionally, future studies should explore moderating factors that may influence the relationship between perceived hope and PTG, such as demographic variables, personality traits, and contextual factors, to understand how these interactions affect PTG levels. Community support, psychological resilience, and external circumstances might play significant roles as well, as indicated by studies like those of Pavlova et al. (2022) and Yurtsenyuk and Sumariuk (2023), which discussed broader socioeconomic and community factors affecting mental health during the war. Additionally, multigroup comparisons and comparative studies across different cultures and conflict situations could provide deeper insights into the universal and culturally specific aspects of how hope influences trauma response to major crises such as the Russia-Ukraine war.

In conclusion, our study suggests hope might represent one of the conditions under which war-induced stress may be transformed into posttraumatic growth rather than becoming a source of persisting psychological distress. If this role of hope is supported by further research, the findings might inspire the development and implementation of new effective approaches to addressing traumatic stress in psychological practice. By recognizing the complex pathways through which trauma impacts individuals, we can better tailor interventions to support recovery, growth, and resilience in the face of adversity. Given the significant role of hope in promoting PTG, interventions in conflict-affected regions should include psychological support that fosters hope alongside traditional mental health services. Programs that encourage community solidarity, highlight positive narratives, and promote future-oriented thinking could be particularly effective. For mental health professionals and policymakers, these insights emphasize the importance of supporting not only direct interventions for anxiety and stress but also fostering the conditions that promote hope, gratitude, and spirituality as resilience factors in the face of global crises.

## Data availability statement

The data analyzed in this study is subject to the following licenses/restrictions: The raw data supporting the conclusions of this article will be made available by the first author upon request. Requests to access these datasets should be directed to AS, alena.slezackova@med.muni.cz.

## Ethics statement

The studies involving humans were approved by Ethics Committee of the Faculty of Medicine, Masaryk University (No 89/2022). The studies were conducted in accordance with the local legislation and institutional requirements. The participants provided their written informed consent to participate in this study.

## Author contributions

AS: Conceptualization, Data curation, Investigation, Project administration, Resources, Writing – original draft, Writing – review & editing, Methodology. TM: Data curation, Formal analysis, Methodology, Validation, Writing – original draft, Writing – review & editing. KM: Conceptualization, Methodology, Writing – original draft, Writing – review & editing, Formal analysis. MS: Writing – review & editing, Writing – original draft. AK: Investigation, Methodology, Writing – original draft, Writing – review & editing.

## References

[ref1] Ai A. Cascio T. Santangelo L. Evans-Campbell T. (2005). Hope, meaning, and growth following the September 11, 2001, terrorist attacks. J. Interpers. Viol. 20, 523–548. doi: 10.1177/0886260504272896 15788553

[ref2] Alpay E. H. (2024). The moderator roles of depression and anxiety symptoms in the relationship between posttraumatic stress disorder and posttraumatic growth in Syrian refugees. Psychol. Trauma 16, 68–75. doi: 10.1037/tra0001613 37917450

[ref3] Barchielli B. Cricenti C. Gallè F. Sabella E. A. Liguori F. Molin D.. (2022). Climate changes, natural resources depletion, COVID-19 pandemic, and Russian-Ukrainian war: what is the impact on habits change and mental health? Int. J. Environ. Res. Public Health 19:11929. doi: 10.3390/ijerph191911929, PMID: 36231229 PMC9565033

[ref4] Barlow D. H. (2000). Unraveling the mysteries of anxiety and its disorders from the perspective of emotion theory. Am. Psychol. 55, 1247–1263. doi: 10.1037/0003-066X.55.11.1247, PMID: 11280938

[ref5] Bazargan-Hejazi S. Dehghan K. Chou S. Bailey S. Baron K. Assari S.. (2023). Hope, optimism, gratitude, and well-being among health professional minority college students. J. Am. Coll. Heal. 71, 1125–1133. doi: 10.1080/07448481.2021.1922415, PMID: 34344275 PMC10699496

[ref6] Bovaird J. A. Koziol N. A. (2012). “Measurement models for ordered-categorical indicators” in Handbook of structural equation modeling. ed. Hoyle R. H. (London: The Guilford Press), 495–511.

[ref7] Brewin C. R. Andrews B. Valentine J. D. (2000). Meta-analysis of risk factors for posttraumatic stress disorder in trauma-exposed adults. J. Consult. Clin. Psychol. 68, 748–766. doi: 10.1037/0022-006X.68.5.748, PMID: 11068961

[ref8] Cann A. Calhoun L. G. Tedeschi R. G. Taku K. Vishnevsky T. Triplett K. N.. (2010). A short form of the posttraumatic growth inventory. Anxiety Stress Coping 23, 127–137. doi: 10.1080/10615800903094273, PMID: 19582640

[ref9] Chaaya C. Devi Thambi V. Sabuncu Ö. Abedi R. Osman Ahmed Osman A. Uwishema O.. (2022). Ukraine - Russia crisis and its impacts on the mental health of Ukrainian young people during the COVID-19 pandemic. Ann. Med. Surg. 79:104033. doi: 10.1016/j.amsu.2022.104033 PMC9221679 35765517

[ref10] Chang M. Kim Y. (2020). The relationship between self-efficacy and posttraumatic growth: mediating effect of cognitive flexibility and hope. J. Korea Content Assoc. 20, 131–141. doi: 10.5392/JKCA.2020.20.06.131

[ref11] Chen Z. J. Bechara A. O. Cowden R. G. Worthington E. L. Jr. (2022). Perceived posttraumatic growth after interpersonal trauma and subsequent well-being among young Colombian adults: a longitudinal analysis. Front. Psychol. 13:993609. doi: 10.3389/fpsyg.2022.993609, PMID: 36405125 PMC9667110

[ref12] Chudzicka-Czupała A. Hapon N. Chiang S. K. Żywiołek-Szeja M. Karamushka L. Lee C. T.. (2023). Depression, anxiety and posttraumatic stress during the 2022 Russo-Ukrainian war, a comparison between populations in Poland, Ukraine, and Taiwan. Sci. Rep. 13:3602. doi: 10.1038/s41598-023-28729-3, PMID: 36869035 PMC9982762

[ref13] Cohn M. A. Fredrickson B. L. (2009). “Positive emotions” in Oxford handbook of positive psychology. eds. Lopez S. J. Snyder C. R. (Oxford: Oxford University Press), 13–24.

[ref14] Collazo-Castiñeira P. Rodríguez-Rey R. Garrido-Hernansaiz H. Collado S. (2022). Prediction of posttraumatic growth in the face of the COVID-19 crisis based on resilience, posttraumatic stress and social participation: a longitudinal study. Front. Psychol. 13:985879. doi: 10.3389/fpsyg.2022.985879, PMID: 36059760 PMC9430662

[ref15] de Girolamo G. Ferrari C. Candini V. Buizza C. Calamandrei G. Caserotti M.. (2022). Psychological well-being during the COVID-19 pandemic in Italy assessed in a four-waves survey. Sci. Rep. 12:17945. doi: 10.1038/s41598-022-22994-4, PMID: 36289273 PMC9606283

[ref16] Dekel S. Ein-Dor T. Solomon Z. (2012). Posttraumatic growth and posttraumatic distress: a longitudinal study. Psychol. Trauma 4, 94–101. doi: 10.1037/A0021865

[ref17] Diab S. Y. Isosävi S. Qouta S. R. Kuittinen S. Punamäki R. L. (2018). The protective role of maternal posttraumatic growth and cognitive trauma processing in Palestinian mothers and infants: a longitudinal study. Lancet 391:S39. doi: 10.1016/S0140-6736(18)30405-7, PMID: 29553438

[ref18] Durbin J. Watson G. S. (1951). Testing for serial correlation in least squares regression, II. Biometrika 38, 159–178. doi: 10.1093/biomet/38.1-2.159 14848121

[ref19] Eltanamly H. Leijten P. van Rooij F. Overbeek G. (2022). Parenting in times of refuge: a qualitative investigation. Fam. Process 61, 1248–1263. doi: 10.1111/famp.12717, PMID: 34523125 PMC9543259

[ref20] Farran C. J. Herth K. A. Popovich J. M. (1995). Hope and hopelessness: Critical clinical constructs. New York: Sage.

[ref21] Fatahi N. Kakamad K. Babakr Z. Tafran K. Økland Ø. (2021). COVID-19 and its triangle effects on human's well-being: a qualitative research method used to collect appropriate data. Acta Inform. Med. 29, 197–204. doi: 10.5455/aim.2021.29.197-204 34759460 PMC8563050

[ref22] Feldman D. B. Balaraman M. Anderson C. (2018). “Hope and meaning-in-life: points of contact between hope theory and existentialism” in The Oxford handbook of hope. eds. Gallagher M. W. Lopez S. J. (Oxford: Oxford University Press), 353–362.

[ref23] Ferriss S. S. Forrest B. S. (2018). Perspectives of Somali refugees on posttraumatic growth after resettlement. J. Refugee Stud. 31, 626–646. doi: 10.1093/jrs/fey006

[ref24] Frankl V. E. (1992). Man's search for meaning. Boston: Beacon Press.

[ref25] Gallagher M. W. Smith L. J. Richardson A. L. D’Souza J. M. Long L. J. (2021). Examining the longitudinal effects and potential mechanisms of hope on COVID-19 stress, anxiety, and well-being. Cognit. Behav. Ther. 50, 234–245. doi: 10.1080/16506073.2021.1877341, PMID: 33544032

[ref26] Ghosn F. Chu T. Simon M. Braithwaite A. Frith M. Jandali J. (2021). The journey home: violence, anchoring, and refugee decisions to return. Amer. Polit. Sci. Rev. 115, 982–998. doi: 10.1017/S0003055421000344

[ref27] Guse T. Shaw M. (2018). “Hope, meaning in life and well-being among a group of young adults” in Hope for a good life. Results of the Hope-barometer international research program. eds. Krafft A. M. Perrig-Chiello P. Walker A. (Cham: Springer), 63–77.

[ref28] Hammer K. Mogensen O. Hall E. O. (2009). The meaning of hope in nursing research: a meta-synthesis. Scand. J. Car. Sci. 23, 549–557. doi: 10.1111/j.1471-6712.2008.00635.x, PMID: 19453659

[ref29] Hayes A. F. (2022). Introduction to mediation, moderation, and conditional process analysis: A regression-based approach. 3rd Edn. London: The Guilford Press.

[ref30] Ho S. Rajandram R. Chan N. Samman N. Mcgrath C. Zwahlen R. (2011). The roles of hope and optimism on posttraumatic growth in oral cavity cancer patients. Oral Oncol. 47, 121–124. doi: 10.1016/j.oraloncology.2010.11.015, PMID: 21183398

[ref31] Horesh D. Brown A. D. (2020). Traumatic stress in the age of COVID-19: a call to close critical gaps and adapt to new realities. Psychol. Trauma 12, 331–335. doi: 10.1037/tra0000592 32271070

[ref32] Hu L. T. Bentler P. M. (1999). Cutoff criteria for fit indexes in covariance structure analysis: conventional criteria versus new alternatives. Struct. Equat. Model. Multidiscipl. J. 6, 1–55. doi: 10.1080/10705519909540118

[ref33] Hullmann S. Fedele D. Molzon E. Mayes S. Mullins L. (2014). Posttraumatic growth and hope in parents of children with cancer. J. Psychosoc. Oncol. 32, 696–707. doi: 10.1080/07347332.2014.955241, PMID: 25158296 PMC4224605

[ref34] Jawaid A. Gomolka M. Timmer A. (2022). Neuroscience of trauma and the Russian invasion of Ukraine. Nat. Hum. Behav. 6, 748–749. doi: 10.1038/s41562-022-01344-4 35437314

[ref35] Jeftić A. Ikizer G. Tuominen J. Chrona S. Kumaga R. (2021). Connection between the COVID-19 pandemic, war trauma reminders, perceived stress, loneliness, and PTSD in Bosnia and Herzegovina. Curr. Psychol. 42, 8582–8594. doi: 10.1007/s12144-021-02407-x, PMID: 34703194 PMC8531897

[ref36] Kalaitzaki A. Tamiolaki A. Tsouvelas G. (2022). From secondary traumatic stress to vicarious posttraumatic growth amid COVID-19 lockdown in Greece: the role of health care workers' coping strategies. Psychol. Trauma Theory Res. Pract. Policy 14, 273–280. doi: 10.1037/tra0001078, PMID: 34323568

[ref37] Kalaitzaki A. E. Tamiolaki A. Vintila M. (2022). The compounding effect of Covid-19 and war in Ukraine on mental health: a global time bomb soon to explode? J. Loss Trauma 28, 270–272. doi: 10.1080/15325024.2022.2114654

[ref38] Karaman M. A. Vela J. C. Garcia C. (2020). Do hope and meaning of life mediate resilience and life satisfaction among Latinx students? Brit. J. Guid. And Couns. 48, 685–696. doi: 10.1080/03069885.2020.1760206

[ref39] Karanci A. Işıklı S. Aker A. Gül E. Erkan B. Özkol H.. (2012). Personality, posttraumatic stress and trauma type: factors contributing to posttraumatic growth and its domains in a Turkish community sample. Europ. J. Psychotraumat. 3:303. doi: 10.3402/ejpt.v3i0.17303 PMC3402104 22893832

[ref40] Keyes C. L. M. (2005). Mental illness and/or mental health? Investigating the axioms of the complete state model of health. J Consul. Clin. Psychol. 73, 539–548. doi: 10.1037/0022-006X.73.3.539 15982151

[ref41] Kimhi S. Eshel Y. Marciano H. Adini B. (2023). Impact of the war in Ukraine on resilience, protective, and vulnerability factors. Front. Public Health 11:1053940. doi: 10.3389/fpubh.2023.1053940, PMID: 37397735 PMC10311639

[ref42] Kinderman P. Tai S. Pontin E. Schwannauer M. Jarman I. Lisboa P. (2015). Causal and mediating factors for anxiety, depression and well-being. Brit. J. Psychiatry 206, 456–460. doi: 10.1192/bjp.bp.114.147553 25858180

[ref43] Klicperova-Baker M. Urban M. (2023). Democratic spirit, emotions, help, and hope during the russian war against Ukraine: experience from the Czech Republic. Appl. Psychol. 3, 1–19. doi: 10.1111/aphw.12486 37715610

[ref44] Kokun O. (2023). The personal growth resources of the adult population following the first months of the war in Ukraine. Int. J. Psychol. 58, 407–414. doi: 10.1002/ijop.12915, PMID: 37170661

[ref45] Kondratyuk V. Puchalska-Wasyl M. M. (2019). Posttraumatic growth, resiliency, and basic hope in soldiers fighting in eastern Ukraine. Roczniki Psychol. 22, 213–231. doi: 10.18290/rpsych.2019.22.3-2

[ref46] Koubová A. Kimhi S. (2024). Prediction of individual, community and societal resilience in the Czech Republic compared to Slovakia during the war in Ukraine. BMC Public Health 24:583. doi: 10.1186/s12889-024-18075-y, PMID: 38395773 PMC10885445

[ref47] Kraemer H. C. Wilson G. T. Fairburn C. G. Agras W. S. (2002). Mediators and moderators of treatment effects in randomized clinical trials. Arch. Gen. Psychiatry 59, 877–883. doi: 10.1001/archpsyc.59.10.877 12365874

[ref48] Krafft A. M. Bosco J. Chukwuorji C. Choubisa R. Comte S. Fenouillet F.. (2023). “Mastering the COVID-19 pandemic crisis: from anxiety to hope” in Hope across cultures. eds. Krafft A. M. Guse T. Slezackova A. (Cham: Springer), 327–405.

[ref49] Krafft A. M. Guse T. Maree D. (2021). Distinguishing perceived hope and dispositional optimism: theoretical foundations and empirical findings beyond future expectancies and cognition. J. Well-Being Assess. 4, 217–243. doi: 10.1007/s41543-020-00030-4

[ref50] Krafft A. M. Martin-Krumm C. Fenouillet F. (2017). Adaptation, further elaboration, and validation of a scale to measure hope as perceived by people. Assessment 26, 1594–1609. doi: 10.1177/1073191117700724 28372460

[ref51] Krafft A. M. Walker A. M. (2018). “Exploring the concept and experience of hope–theoretical and methodological foundations” in Hope for a good life. Results of the Hope-barometer international research program. eds. Krafft A. M. Perrig-Chiello P. Walker A. (Cham: Springer), 3–19. doi: 10.1007/978-3-319-78470-0_1

[ref52] Kurapov A. Kalaitzaki A. Keller V. Danyliuk I. Kowatsch T. (2023). The mental health impact of the ongoing Russian-Ukrainian war 6 months after the Russian invasion of Ukraine. Front. Psych. 14:1134780. doi: 10.3389/fpsyt.2023.1134780, PMID: 37575573 PMC10412819

[ref53] Kurapov A. Pavlenko V. Drozdov A. Bezliudna V. Reznik A. Isralowitz R. (2022). Toward an understanding of the Russian-Ukrainian war impact on university students and personnel. J. Loss Trauma 28, 167–174. doi: 10.1080/15325024.2022.2084838

[ref54] Lee J. Y. Gallagher M. W. (2018). “Hope and well-being” in The Oxford handbook of hope. eds. Gallagher M. W. Lopez S. J. (Oxford: Oxford University Press), 287–298.

[ref55] Linley P. A. Joseph S. (2004). Positive psychology in practice. New Jersey: John Wiley & Sons.

[ref56] Linley P. A. Joseph S. (2011). Meaning in life and posttraumatic growth. J. Loss Trauma 16, 150–159. doi: 10.1080/15325024.2010.519287

[ref57] Liu Z. Doege D. Thong M. Arndt V. (2020). The relationship between posttraumatic growth and health-related quality of life in adult cancer survivors: a systematic review. J. Affect. Disord. 276, 159–168. doi: 10.1016/j.jad.2020.07.044, PMID: 32697695

[ref58] Long L. Phillips C. Glover N. Richardson A. D'Souza J. Cunningham-Erdogdu P.. (2021). A meta-analytic review of the relationship between posttraumatic growth, anxiety, and depression. J. Happiness Stud. 22, 3703–3728. doi: 10.1007/s10902-021-00370-9

[ref59] Mao W. Agyapong V. (2021). The role of social determinants in mental health and resilience after disasters: implications for public health policy and practice. Front. Publ. Health 9:658528. doi: 10.3389/fpubh.2021.658528, PMID: 34095062 PMC8170026

[ref60] Marciano H. Eshel Y. Kimhi S. Adini B. (2022). Hope and fear of threats as predictors of coping with two major adversities, the Covid-19 pandemic and an armed conflict. Int. J. Environ. Res. Public Health 19:1123. doi: 10.3390/ijerph19031123, PMID: 35162144 PMC8834741

[ref61] Mark K. M. Stevelink S. A. M. Choi J. Fear N. T. (2018). Posttraumatic growth in the military: a systematic review. Occup. Environ. Med. 75, 904–915. doi: 10.1136/oemed-2018-105166 30377257

[ref62] Marujo H. Á. Velez M. J. Gonçalves S. P. Neto L. M. Krafft A. M. Casais M. (2021). The value of hope: validation of the perceived hope scale in the Portuguese population. Curr. Psychol. 42, 7981–7989. doi: 10.1007/s12144-021-02115-6

[ref63] Matos M. McEwan K. Kanovský M. Halamová J. Steindl S. R. Ferreira N.. (2021). The role of social connection on the experience of COVID-19 related posttraumatic growth and stress. PLoS One 16:e0261384. doi: 10.1371/journal.pone.0261384, PMID: 34910779 PMC8673633

[ref64] Mattson E. James L. Engdahl B. (2018). Personality factors and their impact on PTSD and posttraumatic growth is mediated by coping style among OIF/OEF veterans. Milit. Med. 183, e475–e480. doi: 10.1093/milmed/usx201, PMID: 29590428

[ref65] McCullough M. E. (2002). Savoring life, past and present: explaining what hope and gratitude share in common. Psychol. Inq. 13, 302–304.

[ref66] Montgomery G. H. David D. DiLorenzo T. Erblich J. (2003). Is hoping the same as expecting? Discrimination between hopes and response expectancies for nonvolitional outcomes. Pers. Individ. Diff. 35, 399–409. doi: 10.1016/S0191-8869(02)00202-7, PMID: 20390044 PMC2852901

[ref67] Mottola F. Gnisci A. Kalaitzaki A. Vintilă M. Sergi I. (2023). The impact of the Russian-Ukrainian war on the mental health of Italian people after 2 years of the pandemic: risk and protective factors as moderators. Front. Psychol. 14:1154502. doi: 10.3389/fpsyg.2023.1154502, PMID: 37303912 PMC10250742

[ref68] Munroe M. Ferrari M. (2022). Posttraumatic growth to psychological well-being: Coping wisely with adversity. Lifelong Learning Book Series. Cham: Springer.

[ref69] Murphy E. R. (2023). Hope and well-being. Curr. Opin. Psychol. 50:101558. doi: 10.1016/j.copsyc.2023.101558 36822123

[ref70] Nolen-Hoeksema S. Davis C. G. (2002). “Positive responses to loss: perceiving benefits and growth” in Handbook of positive psychology. eds. Snyder C. R. Lopez S. J. (New York: Oxford University Press), 598–607.

[ref71] Nunn B. V. (2005). “Getting clear what hope is” in Interdisciplinary perspectives on hope. ed. Eliott J. (New York: Nova Science), 63–78.

[ref72] O'Connor R. C. Wetherall K. Cleare S. McClelland H. Melson A. J. Niedzwiedz C. L.. (2021). Mental health and well-being during the COVID-19 pandemic: longitudinal analyses of adults in the UK COVID-19 Mental Health & Well-being study. Brit. J. Psychiatry 218, 326–333. doi: 10.1192/bjp.2020.212, PMID: 33081860 PMC7684009

[ref73] Pavlova I. Graf-Vlachy L. Petrytsa P. Wang S. Zhang S. (2022). Early evidence on the mental health of Ukrainian civilian and professional combatants during the Russian invasion. Eur. Psychiatry 65:e79. doi: 10.1192/j.eurpsy.2022.2335, PMID: 36408566 PMC9724216

[ref74] Peterson C. Seligman M. E. (2004). Character strengths and virtues: A handbook and classification. Oxford, UK: Oxford University Press.

[ref75] Pięta M. Rzeszutek M. (2022). Posttraumatic growth and well-being among people living with HIV: a systematic review and meta-analysis in recognition of 40 years of HIV/AIDS. Qual. Life Res. 31, 1269–1288. doi: 10.1007/s11136-021-02990-3, PMID: 34518989 PMC9023429

[ref76] Pleeging E. Burger M. van Exel J. (2021). The relations between hope and subjective well-being: a literature overview and empirical analysis. Appl. Res. Qual. Life 16, 1019–1041. doi: 10.1007/s11482-019-09802-4

[ref77] Plomecka M. Gobbi S. Neckels R. Radzinski P. Skorko B. Lazzeri S.. (2021). Factors associated with psychological disturbances during the Covid-19 pandemic: multi-country online study. JMIR Mental Health 8:e28736. doi: 10.2196/28736, PMID: 34254939 PMC8396308

[ref78] Prati G. Pietrantoni L. (2009). Optimism, social support, and coping strategies as factors contributing to posttraumatic growth: a meta-analysis. J. Loss Trauma 14, 364–388. doi: 10.1080/15325020902724271

[ref79] Raccanello D. Burro R. Aristovnik A. Ravšelj D. Umek L. Vicentini G.. (2024). Coping and emotions of global higher education students to the Ukraine war worldwide. Sci. Rep. 14:8561. doi: 10.1038/s41598-024-59009-3, PMID: 38609468 PMC11014932

[ref80] Riad A. Drobov A. Alkasaby M. A. Peřina A. Koščík M. (2023). Nuclear anxiety amid the Russian-Ukrainian war 2022 (RUW-22): descriptive cross-sectional study. Int. J. Environ. Res. Public Health 20:3551. doi: 10.3390/ijerph20043551, PMID: 36834256 PMC9962827

[ref81] Riad A. Drobov A. Krobot M. Antalová N. Alkasaby M. A. Peřina A.. (2022). Mental health burden of the Russian-Ukrainian war 2022 (RUW-22): anxiety and depression levels among young adults in Central Europe. Int. J. Environ. Res. Public Health 19:8418. doi: 10.3390/ijerph19148418, PMID: 35886269 PMC9318466

[ref82] Richardson A. L. (2023). Hope and anxiety. Curr. Opin. Psychol. 53:101664. doi: 10.1016/j.copsyc.2023.101664 37572550

[ref83] Rosseel Y. (2012). Lavaan: an R package for structural equation modeling and more. Version 0.5–12 (BETA). J. Statist. Softw. 48, 1–36. doi: 10.18637/jss.v048.i02

[ref84] Satheesan S. Hameed N. (2017). Personality traits and post-traumatic growth among females living with HIV/AIDS. Ind. J. Health Wellbeing 8, 1147–1150.

[ref85] Satorra A. Bentler P. M. (1994). “Corrections to test statistics and standard errors in covariance structure analysis.” In Latent variables analysis: Applications for developmental research, ed. von Eye A. Clogg C. C. (Cham: Sage Publications), 399–419.

[ref86] Scharbert J. Humberg S. Kroencke L. Reiter T. Sakel S. ter Horst J.. (2024). Psychological well-being in Europe after the outbreak of war in Ukraine. Nat. Commun. 15:1202. doi: 10.1038/s41467-024-44693-6, PMID: 38378761 PMC10879508

[ref87] Scioli A. Biller H. B. (2009). Hope in the age of anxiety: A guide to understanding and strengthening our most important virtue. New York, NY: Oxford University Press.

[ref88] Senger A. R. Gallagher M. W. (2024). The unique effects of hope and gratitude on psychological distress and well-being in trauma-exposed Hispanic/Latino adults. Psychol. Trauma 16, 488–495. doi: 10.1037/tra0001550, PMID: 37498720

[ref89] Shakespeare-Finch J. Lurie-Beck J. (2014). A meta-analytic clarification of the relationship between posttraumatic growth and symptoms of posttraumatic distress disorder. J. Anxiety Disord. 28, 223–229. doi: 10.1016/j.janxdis.2013.10.005 24291397

[ref90] Sheather J. (2022). As Russian troops cross into Ukraine, we need to remind ourselves of the impact of war on health. BMJ 376:o499. doi: 10.1136/bmj.o499, PMID: 35217578

[ref91] Singh K. Jain A. Kaur J. Junnarkar M. Slezackova A. (2016). Cross-cultural differences on Gunas and other well-being dimensions. Asian J. Psychiatry 24, 139–146. doi: 10.1016/j.ajp.2016.09.001, PMID: 27931898

[ref92] Skwirczyńska E. Kozłowski M. Nowak K. Wróblewski O. Sompolska-Rzechuła A. Kwiatkowski S.. (2022). Anxiety assessment in polish students during the Russian-Ukrainian war. Int. J. Envi. Res. Publ. Health 19:13284. doi: 10.3390/ijerph192013284, PMID: 36293865 PMC9602665

[ref93] Slezackova A. (2017). Hope and well-being: Psychosocial correlates and benefits. Valletta: University of Malta.

[ref94] Slezackova A. Prošek T. Malatincová T. Krafft A. M. (2020). Psychometrické vlastnosti české verze Škály prožívané naděje: faktorová struktura a vnitřní konzistence [psychometric characteristics of the Czech version of the perceived Hope scale: factor structure and internal consistency]. Československá Psychol. 64, 288–305.

[ref95] Snyder C. R. (2000). Handbook of hope: Theory, measures and applications. San Diego, CA: Academic Press.

[ref96] Spitzer R. L. Kroenke K. Williams J. B. W. Löwe B. (2006). A brief measure for assessing generalized anxiety disorder: the GAD-7. Arch. Intern. Med. 166, 1092–1097. doi: 10.1001/archinte.166.10.1092 16717171

[ref97] Stallard P. Pereira A. I. Barros L. (2021). Posttraumatic growth during the COVID-19 pandemic in carers of children in Portugal and the UK: cross-sectional online survey. BJ Psych. Open 7:e37. doi: 10.1192/bjo.2021.1, PMID: 33468270 PMC7844169

[ref98] Stasielowicz L. (2022). Adaptive performance in refugees after trauma: how relevant are posttraumatic stress and posttraumatic growth? Int. J. Workplace Health Manag. 15, 711–727. doi: 10.1108/IJWHM-12-2021-0230

[ref99] Stein J. Y. Levin Y. Bachem R. Solomon Z. (2018). Growing apart: a longitudinal assessment of the relation between posttraumatic growth and loneliness among combat veterans. Front. Psychol. 9:893. doi: 10.3389/fpsyg.2018.00893, PMID: 29930525 PMC5999757

[ref100] Sympson S. C. (2000). “Rediscovering hope: understanding and working with survivors of trauma” in Handbook of hope: Theory, measures, and applications. ed. Snyder C. R. (San Diego, CA: Academic Press), 285–300. doi: 10.1016/B978-012654050-5/50017-8

[ref101] Tedeschi R. G. Calhoun L. G. (1995). Trauma and transformation: Growing in the aftermath of suffering. Cham: Sage Publications, Inc.

[ref102] Tedeschi R. G. Calhoun L. G. (1996). The posttraumatic growth inventory: measuring the positive legacy of trauma. J. Traum Stress 9, 455–471. doi: 10.1002/jts.2490090305, PMID: 8827649

[ref103] Tedeschi R. G. Calhoun L. G. (2004). Posttraumatic growth: conceptual foundations and empirical evidence. Psychol. Inq. 15, 1–18. doi: 10.1207/s15327965pli1501_01

[ref104] Tedeschi R. G. Calhoun L. G. (2006). “Foundations of posttraumatic growth” in Handbook of posttraumatic growth. eds. Tedeschi R. G. Calhoun L. G. (Mahwah, NJ: Lawrence Erlbaum Associates Inc), 3–23.

[ref105] The Lancet Regional Health–Europe (2022). The regional and global impact of the Russian invasion of Ukraine. Lancet Reg. Health Eur. 15:100379. doi: 10.1016/j.lanepe.2022.100379, PMID: 35531494 PMC9072997

[ref106] Ulset V. S. von Soest T. (2022). Posttraumatic growth during the COVID-19 lockdown: a large-scale population-based study among Norwegian adolescents. J. Traum Stress 35, 941–954. doi: 10.1002/jts.22801, PMID: 35182076 PMC9305897

[ref107] Uwishema O. Sujanamulk B. Abbass M. Fawaz R. Javed A. Aboudib K.. (2022). Russia-Ukraine conflict and COVID-19: a double burden for Ukraine's healthcare system and a concern for global citizens. Postgrad. Med. J. 98, 569–571. doi: 10.1136/postgradmedj-2022-141895, PMID: 35654572 PMC9340026

[ref108] Vuorio A. Sajantila A. Kovanen P. T. Budowle B. (2022). Maleficent comrades: war in Ukraine and COVID-19. Disaster Med. Publ. Health Prepare 17:e280. doi: 10.1017/dmp.2022.227, PMID: 36226400

[ref109] Wild N. D. Paivio S. C. (2003). Psychological adjustment, coping, and emotion regulation as predictors of posttraumatic growth. J. Aggress. Maltreat. Trauma 8, 97–122. doi: 10.1300/J146v08n04_05

[ref110] Witvliet C. V. O. Richie F. J. Root Luna L. M. Van Tongeren D. R. (2018). Gratitude predicts hope and happiness: a two-study assessment of traits and states. J. Posit. Psychol. 14, 271–282. doi: 10.1080/17439760.2018.1424924

[ref111] Wu X. Kaminga A. Dai W. Deng J. Wang Z. Pan X.. (2019). The prevalence of moderate-to-high posttraumatic growth: a systematic review and meta-analysis. J. Affect. Disord. 243, 408–415. doi: 10.1016/j.jad.2018.09.023, PMID: 30268956

[ref112] Xu W. Pavlova I. Chen X. Petrytsa P. Graf-Vlachy L. Zhang S. X. (2023). Mental health symptoms and coping strategies among Ukrainians during the Russia-Ukraine war in march 2022. Int. J. Soc. Psychiatry 69, 957–966. doi: 10.1177/00207640221143919, PMID: 36598090

[ref113] Yan S. Yang J. Ye M. Chen S. Xie C. Huang J.. (2021). Posttraumatic growth and related influencing factors in discharged COVID-19 patients: a cross-sectional study. Front. Psychol. 12:658307. doi: 10.3389/fpsyg.2021.658307, PMID: 34122242 PMC8189317

[ref114] Yurtsenyuk O. Sumariuk B. (2023). Impact of the war on the mental health of Ukrainians: factors in formation of neurotic and stress-associated mental disorders. Current state of the issue. Art Med. 26, 248–251. doi: 10.21802/artm.2023.2.26.248

[ref115] Zaliska O. Oleshchuk O. Forman R. Mossialoset E. (2022). Health impacts of the Russian invasion in Ukraine: need for global health action. Lancet 399, 1450–1452. doi: 10.1016/S0140-6736(22)00615-8, PMID: 35367006

[ref116] Zhang X. Wang Y. Lyu H. Zhang Y. Liu Y. Luo J. (2021). The influence of COVID-19 on the well-being of people: big data methods for capturing the well-being of working adults and protective factors nationwide. Front. Psychol. 12:681091. doi: 10.3389/fpsyg.2021.681091, PMID: 34234720 PMC8255381

